# L1 incidence reflects pelvic incidence and lumbar lordosis mismatch in sagittal balance evaluation

**DOI:** 10.1097/MD.0000000000011668

**Published:** 2018-07-27

**Authors:** Sung Hoon Choi, Seung Min Son, Dong-Ho Lee, Choon Sung Lee, Won Chul Shin, Chul Gie Hong, Jung Sub Lee, Chang Ju Hwang

**Affiliations:** a Department of Orthopaedic Surgery, Pusan National University Yangsan Hospital, Pusan National University School of Medicine, Busan; b Department of Orthopedic Surgery, Asan Medical Center, University of Ulsan, College of Medicine, Seoul; c Department of Orthopedic Surgery, Kangwon National University, Chuncheon; d Department of Orthopedic Surgery, Pusan National University School of Medicine, Busan, Korea.

**Keywords:** adult sagittal deformity, lumbar lordosis, pelvic incidence, pelvic incidence and lumbar lordosis mismatch, sagittal balance, sagittal spinopelvic modifier, Scoliosis Research Society-Schwab classification

## Abstract

Retrospective study.

To investigate the radiologic and geometrical association between L1 incidence (L1I) with pelvic incidence/lumbar lordosis (PI/LL) mismatch and T1 incidence (T1I) with PI/LL/thoracic kyphosis (TK) mismatch.

The relationship between PI and LL is not clear, and it might be because of the absence of a direct radiologic parameter to represent PI/LL mismatch. To the best of our knowledge, this is the first report on a direct radiologic parameter for representing PI/LL mismatch.

This study is a retrospective review of 146 patients who underwent anteroposterior and lateral standing radiographs of the whole spine. L1I was defined as the angle between the line perpendicular to the L1 upper endplate and the line connecting the midpoint of the sacral endplate to the center of both femoral heads. T1I was defined as the angle between the line perpendicular to the T1 upper endplate and the line connecting the midpoint of the sacral endplate to the center of both femoral heads. Both were validated using the Pearson correlation coefficient and linear regression analysis.

Radiologically measured L1I and T1I were coterminous with calculated measurements of ΔPI/LL and ΔPI/LL/TK in terms of means and standard deviations, respectively. Excellent correlations were found between L1I and ΔPI/LL, and T1I and ΔPI/LL/TK (*R* = 0.997, *P* < .01; *R* = 0.981, *P* < .01, respectively). In linear regression analysis, the slope and intercept of L1I were 0.991 and −0.041, with a predictability of 99.4% (*R*^2^ = 0.994), and those of T1I were 0.990 and −0.026, with a predictability of 99.0% (*R*^2^ = 0.990), respectively.

L1I and T1I were strongly correlated with PI/LL mismatch and PI/LL/TK mismatch, respectively. L1I and T1I are direct parameters that represent PI/LL mismatch and PI/LL/TK mismatch. They would be useful in analyzing sagittal balance.

Level of evidence: Level 3

## 1 Introduction

Humans have unique bipedalism, which is characterized by a narrow and ergonomic base of support. The pelvic orientation has become vertical and broadened to adapt for this bipedalism.^[1]^ The gravity line falls between posterior to the femoral heads and anterior to the sacrum. Vertically oriented sacrum is tilted anteriorly in the sagittal plane; it is represented radiographically as the sacral slope (SS). Moreover, the relationship between the pelvis and femoral heads is known as pelvic incidence (PI), a morphologic parameter unique to each individual.^[2]^ Of these consequences, humans have developed a unique sagittal spinal curve characterized by cervical lordosis and lumbar lordosis (LL) and thoracic kyphosis (TK) to gaze forward horizontally and maintain proper truncal posture within the cone of economy.^[1,3,4]^ The standing posture of a human being is more ergonomically optimal than that of any other bipedal species, thanks to the articulation between the head, trunk, pelvis, and lower limbs.^[5–7]^ The pelvis, the point at which the 2 lower limbs join the spine, may be considered “the first vertebra or pelvic vertebra” as proposed by Dubousset et al.^[3,8]^

Recent studies have revealed that patients with lumbar degenerative disease are characterized by an anterior translation of sagittal balance and loss of LL with an increase in pelvic tilt (PT).^[9–12]^ Sagittal alignment status has been demonstrated to be an independent predictor of clinical status and outcomes in patients undergoing surgeries for adult spinal deformities, degenerative disc diseases, and degenerative spondylolisthesis.^[6,10,13]^ Barrey et al^[11]^ insisted that the main compensatory mechanisms are cervical hyperlordosis, reduction of TK, hyperextension of adjacent lumbar segments, retrolisthesis of the upper lumbar spine, pelvis retroversion, knee flexion, and ankle extension. The basic concept of these compensatory mechanisms is to restore the anterior translation of the center of the gravity line and to recover the horizontal gaze.

Traditionally, the importance of sagittal alignment was less focused upon than that of coronal alignment in surgical treatment of scoliosis. However, several studies have demonstrated that proper sagittal alignment is a much better determinant of health-related quality of life (HRQOL) and pain in patients with adult sagittal deformity (ASD) and that it determines the outcome of spinal deformity surgery.^[10,14,15]^ Recently, a new classification system has been developed for adult spinal deformity, that is, the Scoliosis Research Society (SRS)-Schwab classification, which incorporates spinal and pelvic parameters with excellent inter- and intra-rater reliability and inter-rater agreement for the curve type and each modifier. The sagittal spinopelvic modifiers of the SRS-Schwab classification significantly correlated with clinical statuses and outcomes in multiple standardized measures of HRQOL.^[16–18]^ Schwab et al^[17]^ recommended the threshold values for severe disability (ODI > 40), including PT 22° or more, sagittal vertical axis (SVA) 47 mm or more, and PI − LL 11° or more.

Several studies reported on the adequate restoration of LL based on PI,^[19–21]^ however, so far, the extent of restoration of LL has not been elucidated definitely, and it might be so because of the absence of a direct radiologic parameter to represent it. Furthermore, the value of PI/LL mismatch requires knowing 2 parameters; therefore, it is an indirect value. To the best of our knowledge, a direct radiologic parameter for representing PI/LL mismatch has not been proposed. The main objective of this study is to introduce L1 incidence (L1I) and T1 incidence (T1I), which are intuitive and direct radiologic parameters to investigate the radiologic and geometrical coincidence of PI/LL mismatch and PI/LL/TK mismatch, respectively.

## 2 Methods

### 2.1 Study design and participants

This study was based on retrospective review of 164 patients who underwent anteroposterior and lateral standing radiographs of the whole spine for clinical purposes at a single institution. This study was approved by our hospital's institutional review board. Inclusion criteria included complete visualization of the skeletal structures of the spine and pelvis, especially the femoral heads, sacrum, and the whole spine from the C2 endplate to upper endplates of the S1. Exclusion criteria included lack of visualization of any of the structures aforementioned, skeletal deformity of the spine and pelvis including lumbosacral transitional vertebra and congenital scoliosis, and previous spine and hip surgery.

### 2.2 Data collection and analysis

Clinical demographic and radiographic data were collected from the electronic medical record system and picture-archiving and communications system, respectively. The radiologic protocol was standardized for all patients. For each subject, long-cassette standing lateral radiographs of the spine and pelvis were obtained on 14- × 17-inch films at a tube-to-film distance of 1.5 m. The subjects were instructed to stand in a comfortable position with the hips and knees fully extended and hands resting on a support.

**Figure 1:**
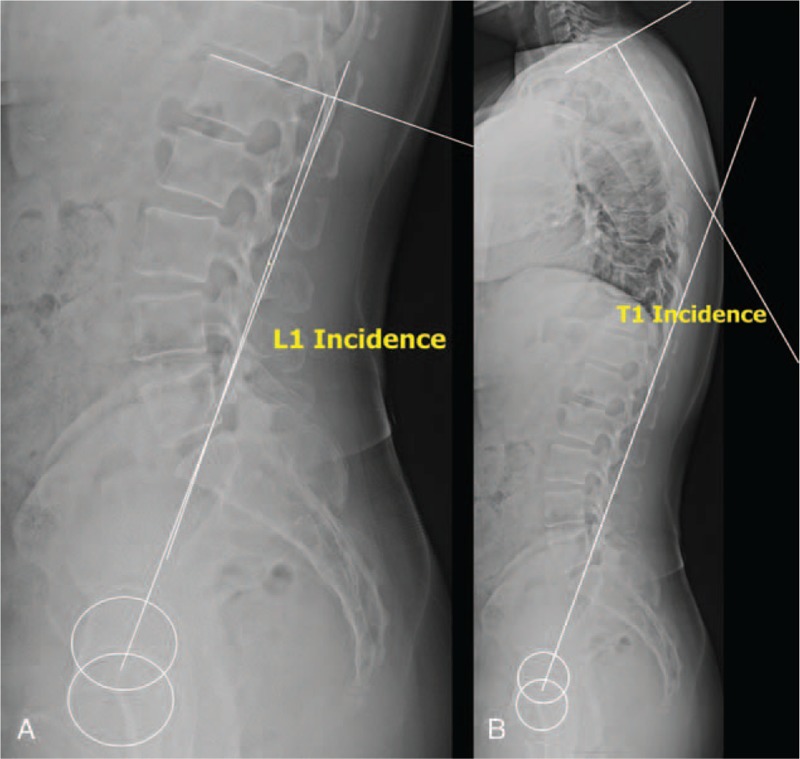
Method used to measure L1 incidence and T1 incidence. L1 incidence was defined as the angle between the line perpendicular to the upper endplate of L1 and the line connecting the midpoint of the sacral endplate to the center of the femoral heads (A). T1 incidence was defined as the angle between the line perpendicular to the upper endplate of T1 and the line connecting the midpoint of the sacral endplate to the center of the femoral heads (B).

L1I was defined as the angle between the line perpendicular to the upper endplate of L1 and the line connecting the midpoint of the sacral endplate to the center of the femoral heads (Fig. 1A). T1I was defined as the angle between the line perpendicular to the upper endplate of T1, and the line connecting the midpoint of the sacral endplate to the center of the femoral heads (Fig. 1B). LL was defined as the angle between the upper endplate of L1 and the upper endplate of S1. TK was defined as the angle between the upper endplate of T1 and the upper endplate of L1. Other sagittal parameters such as PI, SS, PT, LL, L1 slope, TK, T1 slope, and C7 SVA were also measured. PI/LL mismatch (ΔPI/LL) was calculated as the difference between PI and LL (ΔPI/LL = PI – LL), and PI/LL/TK mismatch (ΔPI/LL/TK) was calculated as the sum of PI/LL mismatch and TK [ΔPI/LL/TK = (PI – LL) + TK]. All radiographic parameters were measured by 2 orthopedic spine surgeons who were blinded to the patients’ clinical information. After 3 weeks, the measurements were repeated in a blinded fashion.

### 2.3 Statistical analysis

After descriptive analysis, interobserver reliability and intraobserver reproducibility were assessed according to intraclass correlation coefficients (ICC). The internal consistency of the measurements was characterized as excellent (ICC ≥ 0.9), good (0.7 ≤ ICC < 0.9), acceptable (0.6 ≤ ICC < 0.7), poor (0.5 ≤ ICC < 0.6), or unpredictable (ICC < 0.5). L1I and T1I were validated using Pearson's correlation coefficient and linear regression analysis. Pearson correlation analysis was performed to identify statistically significant correlations between L1I, T1I, and other sagittal parameters including PI, PT, SS, L1I, LL, L1 slope, TK, T1 slope, and C7 SVA. Significant correlation was defined as a correlation with *P* < .05. Linear regression analysis was used to model the relationship between ΔPI/LL and L1I, and ΔPI/LL/TK and T1I. All analyses were performed using SPSS version 21.0 (SPSS, Inc., Armonk, NY, USA).

## 3 Results

Of the 164 patients, 15 patients were excluded due to nonvisualization of skeletal structure, especially the center of the femoral head and the T1 upper endplate, and 3 patients were excluded due to lumbosacral transitional vertebra and congenital scoliosis. A total of 146 patients were enrolled, of which 54 were men (37%) with a mean age of 49.0 ± 16.4 years and 92 were women (63%) with a mean age of 54.9 ± 12.9 years. Demographic data are summarized in Table 1. Radiologically measured L1I and T1I are coterminous with the calculated measurements of ΔPI/LL and ΔPI/LL/TK in terms of means and standard deviations, respectively.

**Table 1:**
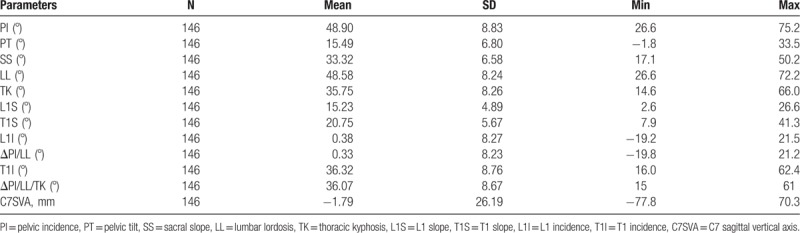
Radiologic measurements of sagittal parameters.

Pearson correlations were assessed between each of the parameters. As shown in Table 2, excellent correlations were found between L1I and ΔPI/LL, and T1I and ΔPI/LL/TK (*R* = 0.997, *P* < .01; *R* = 0.981, *P* < .01, respectively). The values of PI, SS, PT, LL, and TK were similar, and significant correlations were found between each adjacent parameter, which has been reported by previously published studies regarding sagittal profile.^[4]^ Substantial correlations were found between PT and L1I, T1I, and PI (*R* = 0.798, *P* < .01; *R* = 0.751, *P* < .01; *R* = 0.671, *P* < .01, respectively). No correlations were found between C7 SVA and other parameters. In addition, age and sex are not significant factors affecting the correlation between L1I and ΔPI/LL, and T1I and ΔPI/LL/TK (age, *P* = .722, .143; sex, *P* = .375, .166, respectively).

**Table 2:**
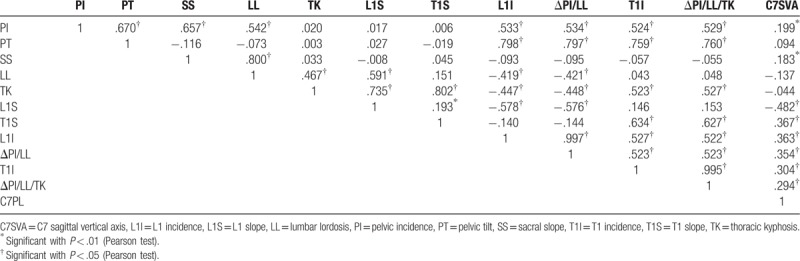
The statistical correlations between each parameters determined using the Pearson correlation coefficient.

**Figure 2:**
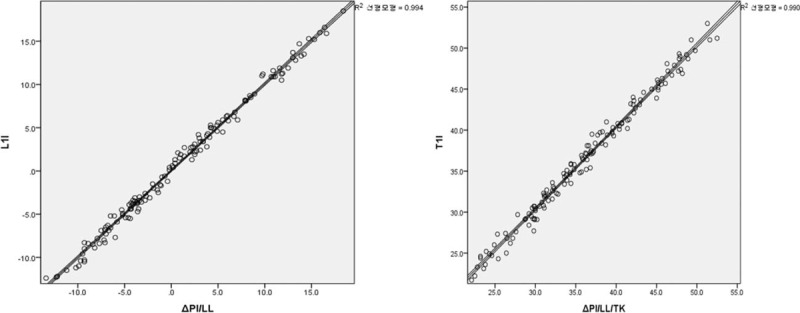
Linear regression analysis between L1 incidence and PI/LL mismatch, and T1 incidence and PI/LL/TK mismatch. The relationship between L1I and PI/LL mismatch showed an equation of the first degree with a slope and an intercept of 0.991 and -0.041, and the relationship between T1I and PI/LL/TK mismatch showed a slope and an intercept of 0.990 and −0.026 with coefficients of determination (*R*^2^) as 0.994 and 0.990, respectively. L1I = L1 incidence, LL = lumbar lordosis, PI = pelvic incidence, T1I = T1 incidence, TK = thoracic kyphosis.

Linear regression analysis was performed to demonstrate the predictability of ΔPI/LL to L1I, and ΔPI/LL/TK to T1I. In the linear regression analysis, the slope of L1I was 0.991, and its intercept was −0.041, with a predictability of 99.4% (*R*^2^ = 0.994) and those of T1I were 0.990 and −0.026, with a predictability of 99.0% (*R*^2^ = 0.990), respectively (Fig. 2). The linear regression equations can be deduced as follows:

Equation 1: L1I = 0.991 × ΔPI/LL −0.041 (*R*^2^ = 0.994)

Equation 2: T1I = 0.990 × ΔPI/LL/TK −0.026 (*R*^2^ = 0.990)

Intraobserver reproducibility was excellent across all radiological parameters. The ICCs of PI, LL, L1I, and T1I were 0.927, 0.932, 0.914, and 0.905, respectively. Interobserver reliability was also excellent for all radiological parameters. The ICCs of PI, LL, L1I, and T1I were 0.919, 0.913, 0.901, and 0.907, respectively.

## 4 Discussion

To realign the lumbosacral imbalance by restoring LL is one of the important goals of spinal fusion surgery, which affects other sagittal spinal balances. Restoration of spinopelvic harmony allows the spine and pelvis to use minimal energy and to maintain posture within the cone of economy. Recent studies have demonstrated that sagittal spinopelvic alignment is a much better determinant of HRQOL and pain in patients with ASD.^[9,14,22]^ The correction of PI/LL mismatch, one of the 3 sagittal spinopelvic modifiers of SRS-Schwab classification, to < 10° results in good clinical outcomes in patients with ASD, flat back deformity, and short-segment transforaminal lumbar interbody fusion.^[17,23]^ Some authors insist that sufficient restoration of LL proportionate to PI is important in spinal surgery, irrespective of the level of fusion.^[14,23–25]^ Patients with spinal deformity and under-corrected sagittal alignment with decreased LL have significantly worse HRQOL scores for physical and social functions, self-image, and pain.^[15,17,26]^ In degenerative lumbar spinal disease, adjacent segment disease risk was increased in the group of patients with PI/LL mismatch ≥ 10° compared with those with PI/LL mismatch < 10°.^[24]^ In addition, higher correction angle in lumbar pedicle subtraction osteotomy is needed in cases of high PT or high PI/LL mismatch.^[27]^ However, Yamada et al. indicated the importance of postoperative PI/LL mismatch, but also noted that 23% of patients achieved good SVA and clinical satisfaction irrespective of inadequate postoperative LL.^[28]^ Inami et al^[21]^ insisted that optimum postoperative PI/LL mismatch is not a fixed value, but depends on the PI. It means that the extent of restoration of LL based on PI has not been elucidated yet. However, further studies might find clues for adequate PI/LL mismatch value through the direct and intuitive parameter, L1I.

In the demographic data, radiographically measured L1I showed very similar values to ΔPI/LL, and T1I to ΔPI/LL/TK, in terms of means and standard deviations. In Pearson correlation analysis, L1I and T1I showed significantly strong correlations with ΔPI/LL and ΔPI/LL/TK (*R* = 0.997, 0.981, and *P* < .01, respectively). Furthermore, in linear regression analysis, the relationship between L1I and ΔPI/LL showed an equation of the first degree with a slope and intercept of 0.991 and −0.041, and the relationship between T1I and ΔPI/LL/TK showed similar values of 0.990 and −0.026 with coefficient of determination (*R*^2^) of 0.994 and 0.990, respectively. Therefore, L1I and T1I almost reflect ΔPI/LL and ΔPI/LL/TK regardless of age and sex, respectively. Interobserver reliability and intraobserver reproducibility of L1I and T1I showed excellent agreement as well.

**Figure 3:**
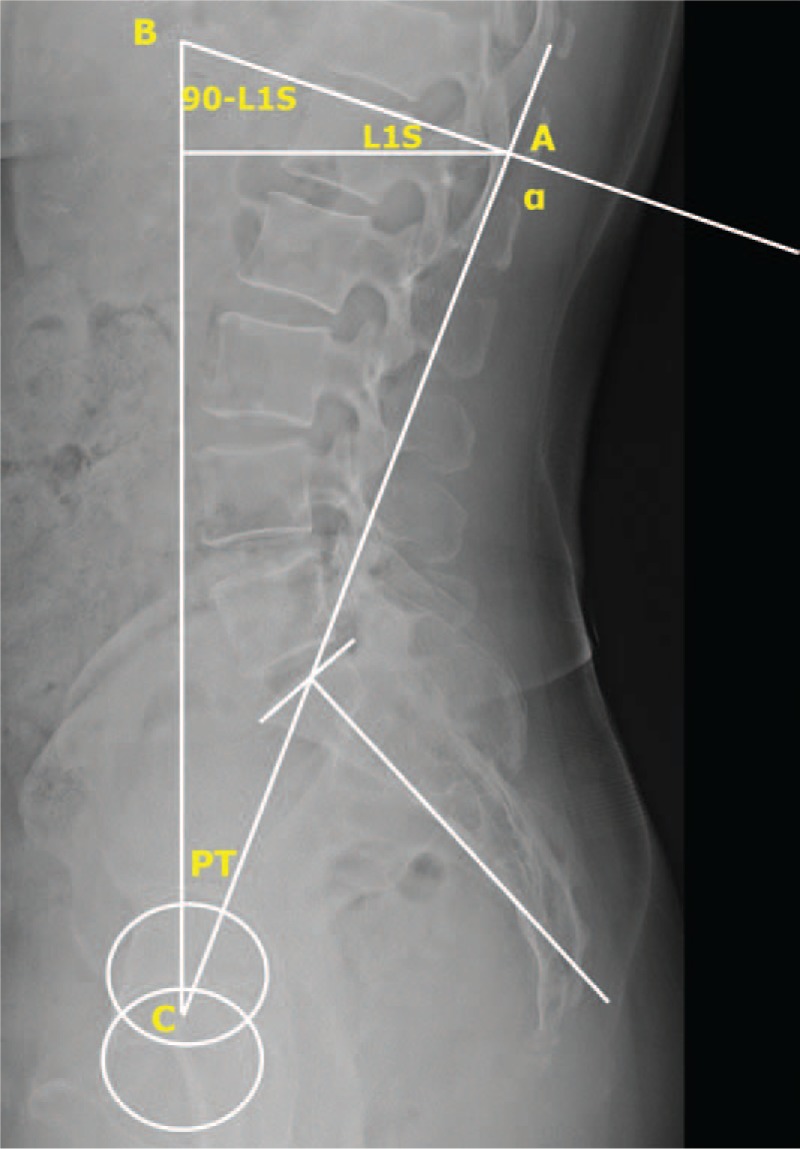
Geometrical proof that L1 incidence represents PI/LL mismatch. In a triangle of ABC, consisting of a line connecting the center of the femoral heads and the center of the sacral upper endplate, an imaginary vertical line, and L1 upper endplate, the angle α is equal to the sum of PT and (90 – L1S) and α is calculated as (90 + PI – LL). Given that L1I is the complementary angle of α, L1I is equal to (PI – LL). L1I = L1 incidence, L1S = L1 slope, LL = lumbar lordosis, PI = pelvic incidence.

This highly accurate conformity of L1I and ΔPI/LL could be drawn geometrically (Fig. 3). For geometrical proof, the 2 prerequisites needed are PI = PT + SS and LL = L1S + SS. In a triangle of ABC, which consisted of a line connecting the center of the femoral heads and center of the sacral upper endplate, an imaginary vertical line, and L1 upper endplate, the angle α is equal to the sum of PT and (90 – L1S), because the size of an exterior angle of a triangle is equal to the sum of the 2 non-neighboring angles (Fig. 3). Through the prerequisites, PT and (90 – L1S) could be translated into (PI – SS) and (90 – LL+SS), respectively. As a result, an angle of α is calculated as (90 + PI – LL). If we define L1I as the complementary angle of α, L1I is equal to (PI – LL).

α = PT + (90 – L1S)

= (PI – SS) + (90 – LL + SS)

= 90 + PI – LL

Therefore, L1I (complementary angle of α) = PI – LL. Similarly, T1I could be deduced as (PI – LL + TK). In fact, L1I and ΔPI/LL and T1I and ΔPI/LL/TK geometrically and statistically represent the same values, and any differences in measurements would be only errors of measurement.

**Figure 4:**
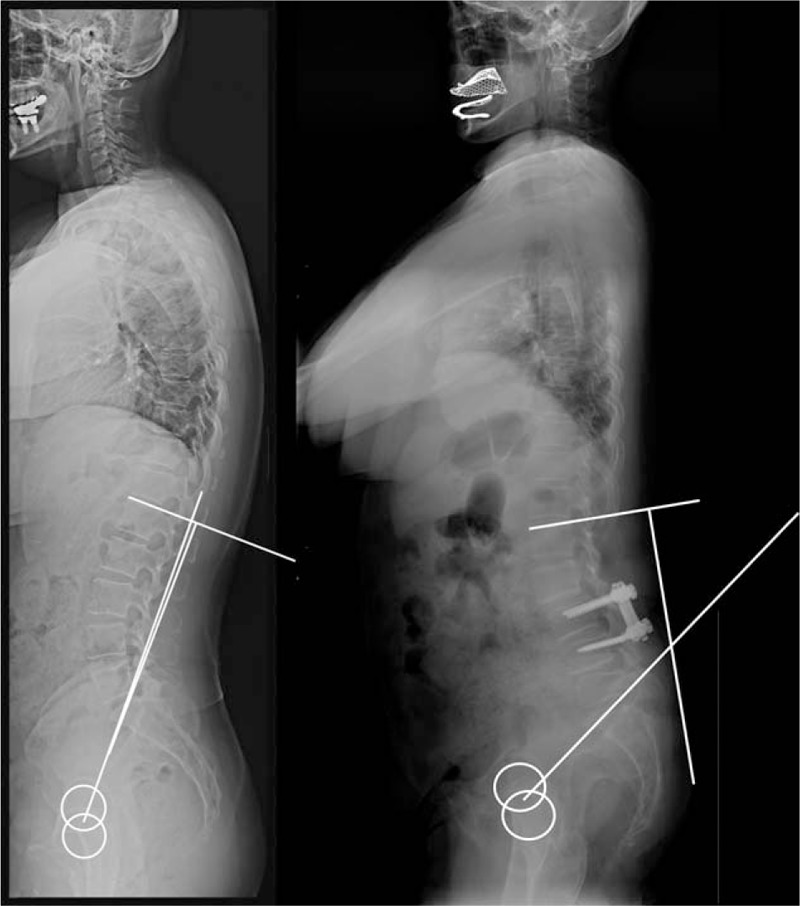
Comparison of a balanced spine and stooped spine according to L1 incidence. In a patient with a balanced spine, the L1I value is almost zero because LL is sufficiently compensated regardless of the amount of PI. On the contrary, in a stooped patient who has sagittal imbalance, L1I is increased in proportion to the PI/LL mismatch, and it is expressed in the whole-spine standing lateral radiograph. L1I = L1 incidence, PI = pelvic incidence, LL = lumbar lordosis.

As can be seen, L1I reflects the PI/LL mismatch intuitively (Fig. 4). In a patient with balanced spine, the value of L1I is almost zero because LL is sufficiently compensated as the amount of PI. On the contrary, in a stooped patient who has sagittal imbalance, L1I is increased in proportion to the PI/LL mismatch and can be seen directly on whole-spine standing lateral radiograph. Therefore, L1I is advantageous as it reflects PI/LL mismatch intuitively by requiring the use of only 2 lines.

Furthermore, L1I could be a referential incidence angle as the inflection point of the spine affected at the thoracolumbar junction. In other words, L1I would not only represent PI/LL mismatch, but it can also be the clue as to where the point should be balanced in proportion to PI in the sagittal plane. Further investigation about the incidence angle of inflection point is warranted.^[5,29]^

Lamartina et al^[5]^ introduced (LL + TK – PI) as a global alignment parameter in their study of the classification of sagittal imbalances based on spinal alignment and compensatory mechanisms. Patients with dominant TK showed a very significant difference in TK – PI/LL mismatch compared with the rest of the patients. They assumed that (TK + LL – PI) represents global alignment considering the amount of loss of LL and the corresponding increase of TK at a given PI. However, this parameter cannot be radiologically expressed; therefore, its implications were not recognized, and the amount of correction of global alignment was unclear. On the contrary, T1I as a positional parameter could represent global alignment, which can be directly seen on radiographs by the relationship of the pelvic, lumbar, and thoracic spine.

Many studies reported on the importance of the effect of T1 slope on cervical spinal alignment. L1I was defined as the sum of the L1 slope and PI, and T1I as the sum of T1 slope and PT. Because it reflects the amount of PT, the concept of incidence of pelvis, lumbar, and thoracic spine represented by PI, L1I, and T1I would be a useful guide to understand and analyze sagittal balance.

Our study has several limitations. First, its retrospective design and relatively small sample size limit its generalizability of the results. Second, this study included patients with various spinal pathologies; therefore, it is not feasible to generalize the values of L1I and T1I as the reference values. Third, this study evaluated only simple standing radiologic parameters, and functional outcomes and HRQOL were not considered. Therefore, the associations between objective measurements, especially L1I and T1I, and clinical outcomes have not been addressed in this study. However, this is the first report to reveal a significant correlation between PI/LL mismatch and L1I, and PI/LL/TK mismatch and T1I. Further large-scale studies on these parameters can help elucidate normal sagittal balance.

In conclusion, L1I and T1I were strongly correlated with PI/LL mismatch and PI/LL/TK mismatch, respectively, and can be the direct parameters representing these mismatches. They would be valuable parameters in analyzing sagittal balance.

## Author contributions

**Conceptualization:** Sung Hoon Choi, Dong-Ho Lee, Choon Sung Lee, Won Chul Shin, Jung Sub Lee.

**Data curation:** Seung Min Son, Won Chul Shin.

**Formal analysis:** Sung Hoon Choi, Chul Gie Hong, Chang Ju Hwang.

**Investigation:** Sung Hoon Choi, Seung Min Son, Chang Ju Hwang.

**Methodology:** Chul Gie Hong, Chang Ju Hwang.

**Project administration:** Choon Sung Lee.

**Software:** Sung Hoon Choi, Won Chul Shin.

**Supervision:** Dong-Ho Lee, Choon Sung Lee, Jung Sub Lee, Chang Ju Hwang.

**Validation:** Seung Min Son, Dong-Ho Lee, Choon Sung Lee, Won Chul Shin, Jung Sub Lee.

**Visualization:** Jung Sub Lee.

**Writing – original draft:** Sung Hoon Choi, Chang Ju Hwang.

**Writing – review & editing:** Sung Hoon Choi, Chul Gie Hong, Chang Ju Hwang.
